# Acute Acalculous Cholecystitis: A Rare Presentation of Primary Epstein-Barr Virus Infection in Adults—Case Report and Review of the Literature

**DOI:** 10.1155/2017/5790102

**Published:** 2017-01-17

**Authors:** Zuhal Yesilbag, Asli Karadeniz, Fatih Oner Kaya

**Affiliations:** ^1^Department of Infectious Diseases and Clinical Microbiology, Bakirkoy Dr. Sadi Konuk Education and Research Hospital, Istanbul, Turkey; ^2^Department of Infectious Diseases and Clinical Microbiology, Maltepe University Faculty of Medicine, Istanbul, Turkey; ^3^Department of Internal Medicine, Maltepe University Faculty of Medicine, Istanbul, Turkey

## Abstract

Primary Epstein-Barr virus (EBV) infection is almost always a self-limited disease characterized by sore throat, fever, and lymphadenopathy. Hepatic involvement is usually characterized by mild elevations of aminotransferases and resolves spontaneously. Although isolated gallbladder wall thickness has been reported in these patients, acute acalculous cholecystitis is an atypical presentation of primary EBV infection. We presented a young women admitted with a 10-day history of fever, nausea, malaise who had jaundice and right upper quadrant tenderness on the physical examination. Based on diagnostic laboratory tests and abdominal ultrasonographic findings, cholestasis and acute acalculous cholecystitis were diagnosed. Serology performed for EBV revealed the acute EBV infection. Symptoms and clinical course gradually improved with the conservative therapy, and at the 1-month follow-up laboratory findings were normal. We reviewed 16 adult cases with EBV-associated AAC in the literature. Classic symptoms of EBV infection were not predominant and all cases experienced gastrointestinal symptoms. Only one patient underwent surgery and all other patients recovered with conservative therapy. The development of AAC should be kept in mind in patients with cholestatic hepatitis due to EBV infection to avoid unnecessary surgical therapy and overuse of antibiotics.

## 1. Introduction

Epstein-Barr virus (EBV) is a member of Herpesviridae family and causes infectious mononucleosis (IM) characterized by sore throat, fever, and lymphadenopathy. IM is common worldwide and is almost always a self-limited disease most commonly seen in young adults. Hepatic involvement is usually characterized by mild elevations of aminotransferases seen in 80–90% of the cases and resolves spontaneously; severe cholestasis and jaundice are rare [1–6]. Acute acalculous cholecystitis (AAC), an inflammatory process of gallbladder in the absence of gallstones, usually occurs in critically ill patients with severe infections or injuries and antibiotic treatment and surgery may be needed [7]. Although isolated gallbladder wall thickness (GWT) or hydrops have been reported in the patients with IM, AAC is an atypical presentation of primary EBV infection [8, 9]. We described a patient with AAC due to acute EBV infection presented with jaundice and successfully treated with conservative therapy and reviewed similar adult cases reported in the literature.

## 2. Case Presentation

A 30-year-old woman was admitted to our hospital with a 10-day history of fever, nausea, and malaise. She had a recent upper respiratory tract infection with sore throat and nonproductive cough and took a beta-lactam antibiotic and paracetamol. She noted that 3 days prior to admission she had realised the jaundice of her sclera. Her past medical history was unremarkable. She had no drug allergies and she had no history of alcohol consumption, blood transfusion, injection drug use, and recent travel. She had no family history of liver disease. On admission she had a body temperature of 39.2°C, pulse rate of 112/min, and respiratory rate of 16/min. Blood pressure was 110/70 mmHg and jaundice of sclera and skin was observed. Tonsil and pharynx were normal. On examination of the abdomen, the right upper quadrant was tender and painful, hepatomegaly was not present, and the spleen was enlarged two fingers below the costal margin. Initial laboratory tests revealed aspartate aminotransferase (AST): 233 (8–37) U/L, alanine aminotransferase (ALT): 220 (15–65) U/L, gamma glutamyl transferase (GGT): 471 (5–55) U/L, alkaline phosphatase (ALP): 376 (50–136) U/L, lactate dehydrogenase (LDH): 909 (81–234) U/L, C-reactive protein (CRP): 13.6 (0–0.5) mg/dL, erythrocyte sedimentation rate (ESR): 34 (0–15) mm/h, total bilirubin: 15.4 (0.0–1.0)  mg/dL and direct bilirubin: 14.5 (0.0–0.3)  mg/dL, white blood cell (WBC) count: 8.7 × 10^3^ (4.5–11 × 10^3^)/mm^3^ with 66.4% neutrophils, 27.1% lymphocytes, and 5.6% monocytes, hemoglobin (Hb): 10.4 (11.7–15.5) g/dL, hematocrit: 30.6 (35–45)%, and platelets: 121 × 10^3^ (150–450 × 10^3^)/mm^3^. Bacterial cultures of urine and blood were negative. Chest X-ray did not show any pathologic changes. Serological markers for hepatitis A, B, and C viruses, anti-HIV, CMV IgM, toxoplasma IgM, anti-nuclear antibody, anti-mitochondrial antibody, smooth muscle antibody, anti-dsDNA, and ANCA tests were negative. Brucella agglutination and Gruber Widal tests were negative. Serology performed for EBV revealed the acute EBV infection: IgM and IgG antibodies against viral capsid antigen (VCA) were positive whereas IgG antibodies against Epstein-Barr nuclear antigen (EBNA) were negative. An ultrasound scan of the upper abdomen showed a thickened and oedematous gallbladder wall (7.4 mm) with pericholecystic and perihepatic fluid and absence of cholelithiasis or dilatation of the biliary tract, suggesting an acute acalculous cholecystitis. On the 4th day of hospital stay, AST, ALT, total bilirubin, and direct bilirubin increased to 469 U/L, 361 U/L, 19.1 mg/dL, and 17.7 mg/dL, respectively, and ALP and GGT decreased to 289 U/L and 308 U/L, respectively. During admission hemolysis developed with a drop in Hb from 10.4 to 8.6 g/dL. After the 5th day of hospital stay, aminotransferase and bilirubin levels began to decrease; ALP and GGT levels continued to fall which were already decreasing since the first day of admission. Platelets were low on the day of admission (121 × 10^3^/mm^3^) and rose to normal level on the 4th day. We did not use antibiotic and because symptoms and clinical course gradually improved with the conservative therapy, percutaneous drainage was thought to be unnecessary. Abdominal pain and fever resolved, and the patient was discharged on the 7th day of admission. At the 1-month follow-up, the patient was asymptomatic, jaundice had resolved, and laboratory findings were normal.

## 3. Discussion

AAC is defined as acute inflammatory process of the gallbladder without evidence of gallstones and it contributes to 5–10% of overall cholecystitis in adults. It is usually described in patients with abdominal trauma, extensive burns, long term total parenteral nutrition, and systemic diseases including systemic lupus erythematosus and Kawasaki disease [24, 25]. It has also been reported during several infections such as viral hepatitis A, cytomegalovirus,* Salmonella *spp., malaria infections [26]. Although hepatitis with mild elevations in serum aminotransferases is a common characteristic of EBV infection, AAC is rare in the course of primary EBV infection.

Reviewing the literature, we found 16 adult cases (including the present case) of AAC due to primary EBV infection published between 2007 and 2016 (Table 1) [10–23]. It has been noticed that almost all cases were seen in females (15 out of 16). Eicosanoid proinflammatory mediators also play a role in AAC and eicosanoid synthesis depends on estrogen levels, suggesting a probable relationship between estrogen and the development of disease [27]. AAC can be seen at any age of people, but it is most commonly seen in the fourth or eighth decades of life [28]. Although AAC represents 30–50% of all cases of acute cholecystitis in pediatric population, it is rare in childhood compared with the adults. Most of the cases we reviewed were younger than 25 years. This can be explained by the fact that EBV infections are most commonly seen in young adults. Most of the reported cases occurred in Europe, and 2 of the cases were from our country.

Cholestasis, increased bile viscosity, ischemia, and secondary infections were described in the pathogenesis of AAC [22]. The mechanism of EBV-associated AAC is not clear. Most of the authors hypothesized that EBV-induced hepatitis is a cause of cholestasis, inducing gallbladder inflammation and AAC [20]. We had documented elevations in cholestatic markers in 14 of 16 cases (Table 1). Our patient had cholestatic hepatitis with jaundice and markedly elevations in gama-glutamyltransferase, alkaline phosphatase and bilirubin levels without biliary obstruction. Therefore, it could be postulated that EBV-associated cholestasis induced gallbladder inflammation and the development of AAC in these patients. In hepatitis A infection, direct viral invasion of the gallbladder has been documented by detection of viral antigen in most epithelial cells of the gallbladder [13]. However EBV is known to infect oral epithelial cells, but direct invasion of the gallbladder wall has not been described [22]. The presence of Gilbert's syndrome in children with EBV-associated cholestasis could also play a role in the development of AAC [29].

The main clinical symptoms of AAC are represented by fever, vomiting, and right upper quadrant pain. In our case, the patient was admitted with fever, nausea, malaise, and jaundice, and also the right upper quadrant was tender and painful. The classical symptoms of EBV infection such as sore throat, pharyngitis, and lymphadenopathy have been reported in only three patients (9 with missing information), six patients (4 with missing information), and eight patients (2 with missing information), respectively. However all of the patients had abdominal symptoms and almost all cases had positive Murphy's sign (1 with missing information).

14 of the patients had ALT elevations (2 with missing information), 13 patients had markedly elevated bilirubin levels and 14 patients had high ALP or GGT levels (2 with missing information). In a retrospective study Vine et al. recently showed that EBV was the causative agent in only 0.85% of 1995 adult patients with jaundice and hepatitis and the increase of liver enzymes did not exceed 1400 IU/L, and more profound impairment of liver enzymes usually suggests other causes [30]. In our review the mean ALT level was 281 IU/L, mean AST level was 253 IU/L, and the highest increase in ALT was in Cholongitas's study (584 IU/L). Severe liver injury during EBV infection is very rare and is mostly reported in the posttransplant and immunodeficiency settings [30]. Markers of severe involvement such as hyperammonemia or prolonged prothrombin time were not documented in any of the cases we reviewed.

The diagnosis of AAC is based on clinical symptoms, laboratory tests, and ultrasonographic image. Abdominal ultrasonography is the main diagnostic method. Criteria such as GWT of over 3.5 mm, distention of the gallbladder, localized tenderness (sonographic Murphy's sign), and pericholecystic fluid and sludge have been used for the diagnosis of AAC. The combination of two or more of the above-mentioned findings, in the appropriate clinical setting, is considered to be diagnostic [7, 31]. Deitch and Engel reported a specificity of 90% with a 3 mm GWT and 98,5% with a 3.5 mm GWT, whereas sensitivity was 100% at 3 mm but only 80% at 3.5 mm [32, 33]. According to this, a GWT of 3.5 mm or more is generally accepted to be diagnostic for AAC. In our case the GWT was 7.4 mm with a pericholecystic fluid and GWT of other cases we reviewed were between 4.5 and 16 mm. Isolated GWT is a well-known feature during IM and has been been proposed as a sign of severity of the illness [8, 34]. However we consider that the wall thickening of our patient's gallbladder was a consequence of the developed AAC.

The treatment of AAC involves serial examinations, gallbladder ultrasonography, and cholecystectomy when indicated by deteriorating clinical or ultrasonographic findings. AAC due to bacterial infections should be severe and treated with antibiotics, and gallbladder percutaneous drainage should be performed [7]. In our case percutaneous drainage was thought to be unnecessary, because her symptoms and clinical course were gradually improved with conservative therapy. The treatment of EBV-associated AAC is usually supportive and antibiotics are not indicated and most cases resolve spontaneously. All the cases we reviewed had favourable outcomes. In most of the cases, antibiotics were discontinued after the diagnosis of EBV infection was performed. There was no difference in clinical course of the patients treated with antibiotics compared with patients not receiving antibiotics in the cases we reviewed. We did not use antibiotics in our case. The patient was admitted to our hospital with a history of beta-lactam antibiotic usage for the last 7 days, but her symptoms gradually increased despite antibiotic usage. Therefore we wanted to see the results of viral markers and the 3rd day of hospital stay primary EBV infection was investigated. While AAC is considered as a surgical emergency in critically ill patients, in the course of EBV-associated AAC surgical intervention is rarely necessary. In our review, only one patient, receiving azathioprine for an inflammatory bowel disease, reported by Hagel et al. underwent cholecystectomy [15]. Although EBV infections are rarely severe, AAC may cause gallbladder perforation [20]. Thus, a 22-year-old woman with severe EBV-associated AAC with suspected gallbladder perforation was described by Chalupa et al. and was treated without surgery [14].

In summary we reviewed sixteen adult cases with AAC associated with primary EBV infection reported in the literature since 2007. All but one case were females and most of them were young adults. In general all patients had similar gastrointestinal symptoms (classic symptoms of EBV infection were not predominant), and markedly elevations in cholestatic markers and moderate transaminase level elevations were the most common laboratory findings. GWT in the ultrasound scanning were between 4.5 and 16 mm. Only one patient required surgical intervention and all other patients recovered with conservative therapy even though one was suspected to have perforation of the gallbladder.

## 4. Conclusion

Acute acalculous cholecystitis may develop during the course of primary EBV infection, especially in young women with cholestatic hepatitis. Abdominal ultrasonography should be performed when a patient is admitted with right upper quadrant pain or jaundice during the course of IM, to avoid unnecessary surgical therapy and overuse of antibiotic.

## Figures and Tables

**Table 1:** Characteristics of reported cases of AAC due to primary EBV infection in adults.

Authors	Age, sex	Country	Sore throat	Pharyngitis	Lymph-adeno-pathy	Murphy's sign positive	Hepato-spleno-megaly	AST (IU/l)	ALT (IU/l)	Total bilirubin	ALP (IU/l)	GGT (IU/L)	GWT	Antibiotics
Koch et al. (2007) [10]	53, F	Netherlands	—	—	—	—	—	—	339	120 *µ*mol/L (0–17)	1081 (40–120)	—	10 mm	No
Iaria et al. (2008) [11]	18, F	Italy	Yes	Yes	Yes	Yes	Yes	220 (5–45)	328 (5–45)	7 mg/dL (0–1.9)	312 (38–148)	142 (7–32)	9 mm	Yes
Cholongitas et al. (2009) [12]	19, F	Greece	Yes	Yes	Yes	Yes	—	426 (<40)	584 (<40)	6.5 mg/dL	710 (<280)	156 (<50)	8 mm	No
Yang et al. (2010) [13]	20, F	Korea	Yes	Yes	Yes	Yes	Yes	171	299	0.7 mg/dL	727	202		Yes
Chalupa et al. (2009) [14]	22, F	Czech Republic	—	Yes	Yes	Yes	Yes	6.87 *µ*kat/L (0–0.65)	12.79 *µ*kat/L (0–0.8)	143 *µ*mol/L (0–20)	2.2 *µ*kat/L (0.5–2)	3.06 *µ*kat/L (0–0.6)	6 mm	Yes
Hagel et al. (2009) [15]	22, F	Germany	—	—	—	Yes	Spleno-megaly	—	—	254 *µ*mol/L	—	—	7 mm	Yes
Beltrame et al. (2012) [16]	29, F	Italy	—	Yes	Yes	Yes	—	121 (10–35)	166 (10–35)	23.2 *µ*mol/L (1.7–17)	161 (53–151)	145 (3–45)	15 mm	Yes
Nagdev and Ward (2011) [17]	18, F	California	No	No	No	Yes	—	118	—	1.2 mg/dL	146	—	>10 mm	Yes
Dylewski (2012) [18]	22, F	Canada	—	No	No	Yes	Spleno-megaly	—	89	Normal	—	—	5 mm	Yes
Carrascosa et al. (2012) [19]	22, F	Spain	—	—	No	Yes	Yes	329	464	43 *µ*mol/L	239	—	14 mm	No
Gagneux-Brunon et al. (2014) [20]	18, F	France	—	No	Yes	Yes	No	321	214	20 *µ*mol/L	165	64	12 mm	Yes
Gagneux-Brunon et al. (2014) [20]	20, F	France	—	No	Yes	Yes	No	453	494	38 *µ*mol/L	133	286	16 mm	Yes
Çelik et al. (2014) [21]	48, F	Turkey	—	—	Yes	Yes	—	221 (5–35)	165 (5–40)	14.43 mg/dL (0.1–1)	516 (90–260)	224 (7–32)	Marked thickened	Yes
Agergaard and Larsen (2015) [22]	34, F	Denmark	No	Yes	No	Yes	No	—	61 (10–45)	42 *µ*mol/L (5–25)	429 (35–105)	—	11.3 mm	Yes
Koufakis and Gabranis (2016) [23]	21, M	Greece	No	No	No	Yes	—	172 (<40)	232 (<40)	6.31 mg/dL (0-1)	179 (<140)	350 (<30)	4.5 mm	No
Present Case	30, F	Turkey	No	No	Yes	Yes	Spleno-megaly	233 (8–37)	220 (15–65)	15.4 mg/dL (0-1)	376 (50–136)	471 (5–55)	7.4 mm	No

ALT: alanine aminotransferase, AST: aspartate aminotransferase, ALP: Alkaline phosphatase, GGT: gamma-glutamyl transferase, GWT: gallbladder wall thickness, (): normal values only noted when described in the study, and —:missing values.
